# Algebraic constraints and algorithms for common lines in cryo-EM

**DOI:** 10.1017/S2633903X24000072

**Published:** 2024-05-16

**Authors:** Tommi Muller, Adriana L. Duncan, Eric J. Verbeke, Joe Kileel

**Affiliations:** 1 Mathematical Institute, University of Oxford, Oxford, UK; 2 Department of Mathematics, University of Texas at Austin, Austin, TX, USA; 3 Program in Applied and Computational Mathematics, Princeton University, Princeton, NJ, USA; 4 Oden Institute, University of Texas at Austin, Austin, TX, USA

**Keywords:** ADMM, common lines, cryo-electron microscopy, discrete heterogeneity, group synchronization, low-rank matrix

## Abstract

We revisit the topic of common lines between projection images in single-particle cryo-electron microscopy (cryo-EM). We derive a novel low-rank constraint on a certain 2*n* × *n* matrix storing properly scaled basis vectors for the common lines between *n* projection images of one molecular conformation. Using this algebraic constraint and others, we give optimization algorithms to denoise common lines and recover the unknown 3D rotations associated with the images. As an application, we develop a clustering algorithm to partition a set of noisy images into homogeneous communities using common lines, in the case of discrete heterogeneity in cryo-EM. We demonstrate the methods on synthetic and experimental datasets.

## Impact Statement

Single-particle cryo-electron microscopy (cryo-EM) is an imaging technique used to determine the 3D structure of biomolecules from noisy 2D projection images. This article revisits one of the first approaches to cryo-EM image processing, namely common lines between pairs of 2D class averages coming from the Fourier slice theorem. We present a novel mathematical approach for dealing with common lines: in contrast to some alternatives, it operates directly on the common lines themselves and avoids triplewise angular reconstitution completely. The article then derives novel algebraic constraints on sets of consistent common lines, including a straightforward low-rank matrix condition. The algebraic conditions are incorporated into optimization methods arising from the field of computer vision to produce new methods for computational tasks involving common lines. In particular, we achieve improved accuracy in common line denoising and rotation recovery at low signal-to-noise ratios. We also present a method to detect homogeneous communities of 2D class averages in the case of a cryo-EM dataset with multiple molecular conformations. Altogether this work clarifies a classic topic in cryo-EM, and opens the door to applying common lines techniques on more challenging datasets.

## 1. Introduction

Single-particle cryo-electron microscopy (cryo-EM) is an imaging technique capable of recovering the high-resolution 3D structure of molecules from many noisy tomographic projection images taken at unknown viewing angles. One of the first approaches for 3D reconstruction, known as angular reconstitution, is based on the common line property of projection images induced by the Fourier slice theorem.^(^^1^^,^^2^^)^ Due to the low signal-to-noise ratio (SNR) in cryo-EM data, detecting common lines is a difficult task^(^^3^^)^: even today when applied to denoised averages of images, referred to as 2D class averages. Detecting common lines is subject to angular errors and incorrectly identified common lines. Although methods which seek to minimize global errors in the estimated viewing directions have increased the utility of common lines methods,^(^^4^^)^ additional constraints on common lines are needed to improve their accuracy and robustness.

In this article, we propose a novel approach for dealing with common lines. Specifically, we assemble the estimated common lines for a dataset of 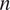 images into a certain 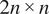 matrix, which stores properly scaled basis vectors for the common lines (Theorem 3.2). The matrix directly encodes common lines data, without requiring angular reconstitution on various subsets of images or needing voting procedures like some existing formulations.^(^^3^^,^^5^^)^ As such, it yields a direct and more global approach than prior constructions for common lines.

As a main contribution, we derive algebraic constraints on the matrix of common lines, which must be satisfied in order for a set of common lines to be consistent with a single asymmetric molecular conformation. The constraints include a straightforward low-rank condition on the matrix, as well as various sparse quadratic constraints. Importantly, the constraints enable new strategies for computational tasks involving common lines, in particular for denoising common lines; estimating 3D rotations; and clustering heterogeneous image sets into homogeneous subsets. We demonstrate this by adapting optimization algorithms from other domains to these tasks, using the algebraic constraints. We remark that our constraints seem better suited for numerical optimization than the semialgebraic constraints found in prior work.^(^^6^^)^

Notably, the clustering problem is a recent application of common lines.^(^^7^^)^ In more detail, the goal is to sort discretely heterogeneous image sets of multiple molecules into communities corresponding to homogeneous image subsets. This application is motivated by the increasing complexity of cryo-EM datasets, where samples may not be purified and thus the number of distinct molecules contained in a dataset is more than one.^(^^8^^–^^10^^)^ Our algebraic constraints and optimization algorithms enable consistency checks of subsets of images, to test whether the subset corresponds to a single molecule.

As a mathematical guarantee, we prove that computing the correct scales in the homogeneous case admits an essentially unique global optimum, see Theorem 5.1. We implement our algorithms and test them on simulated and real datasets in Section 7. The results demonstrate that our methods can be successful when applied to 2D class averages at noise levels comparable to experimental data, in both the homogeneous and discretely heterogeneous cases. We conclude with a discussion of potential future improvements.

### 1.1. Advantages

There are several advantages to our approach for dealing with common lines:

- The new formulation is directly in terms of the data, that is, in terms of the common lines themselves.
- It involves multiple common lines simultaneously and does not require triplewise angular reconstitution at all (in contrast to (5) for instance), making our approach fully global and potentially more robust to noise than alternatives.
- The algebraic constraints can be incorporated into existing optimization algorithms that have seen success in computer vision applications.^(^^11^^)^
- The resulting algorithms outperform existing methods for denoising and rotation recovery on noisy simulated data, and perform comparably well for clustering heterogeneous image sets on real data, even though the optimization algorithms are off-the-shelf.

## 2. Background

First, we recall a standard simplified mathematical model for cryo-EM, in the homogeneous case of one molecular conformation. We assume there exists a 3D function 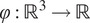 describing the electrostatic potential generated by the molecule. As data, we receive 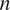 two-dimensional tomographic projection images, denoted 
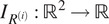
 for 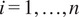 , where 
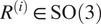
 are 3D rotations associated with each image. The goal of single-particle cryo-EM is to recover the underlying 3D structure 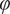 from the set of 2D tomographic projection images which are observed at unknown rotations. The images, in their idealized and noiseless form, have the following Fourier transforms due to the Fourier slice theorem:(1)

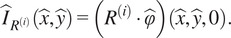

Here 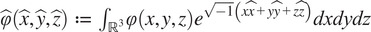 denotes the Fourier transform of 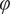 , and 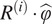 denotes the rotation of 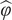 by 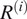 . Writing 
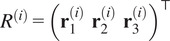
 , (1) reads(2)

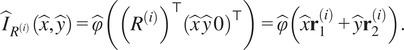

Generically, for asymmetric molecules 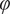 and distinct rotations 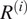 and 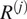 , there exist unique lines through the origin in the domain of the Fourier-transformed images 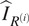 and 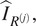 respectively 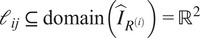 and 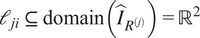 , such that the restrictions(3)

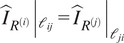

are equal as functions on 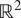 . In cryo-EM, one says that 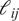 and 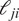 are the *common lines* between the 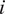 th and 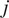 th image. In modest-noise settings, which are arrived at by working with 2D class averages instead of raw tomographic images,^(^^12^^)^ common lines can be estimated from real cryo-EM data. They give basic ways to do 3D reconstruction in cryo-EM; for example, see the angular reconstitution technique of van Heel^(^^2^^)^ or the works of Shkolnisky, Singer, and their collaborators^(^^3^^–^^5^^)^ for example.

From (2), the common lines 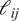 and 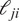 may be found mathematically by expressing the single line in 3D space:(4)

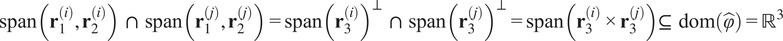

in the coordinate system of the *i*th and *j*th images, respectively. Here, 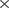 denotes the cross product in 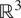 . Combining (2) and (4), (3) may be written as:

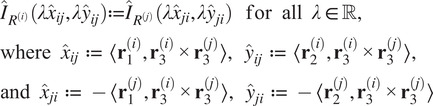

where 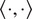 denotes the standard inner product in 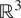 . Common lines can therefore be encoded via:

Definition 2.1. Vectors 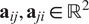 are called *representatives* for the common lines 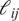 and 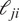 if there exists a nonzero scalar 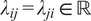 such that(5)

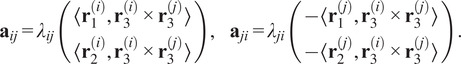

Equivalently, representatives 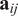 and 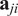 are choices of basis vectors for the common lines 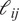 and 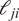 which satisfy 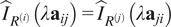 for all 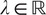 . Representatives for common lines can be estimated from 2D class averages in practice.

We stress that, although quite standard, the model (1) is greatly simplified. It neglects the effects of contrast transfer functions (CTFs), imperfect centering in particle picking, and blurring in class averaging. Further, we have restricted attention to the case of asymmetric molecules, as otherwise common lines are only unique up to the action of the relevant symmetry group (e.g., see (13)).

## 3. Constraints on sets of common lines

### 3.1. The common lines matrix

We introduce an object to keep track of all common lines in a dataset. It is the main object of this article.

Definition 3.1. A *common lines matrix* associated to rotations 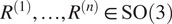 is a matrix 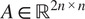 , which when regarded as an 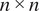 block matrices with 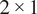 blocks 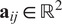 is such that 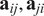 are representatives for the common lines 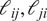 if 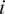 and 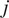 are distinct and 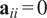 otherwise. If the scalars 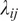 in (5) are all equal to 1, then we call 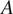 the *pure common lines matrix.*

Thus a common lines matrix 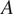 associated to 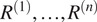 is uniquely defined up to 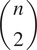 nonzero real scalars 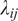 ( 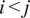 ). In real data settings where the 2D class averages are sufficiently denoised, we can estimate 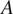 from data by estimating representatives for the common lines.

We now present constraints which a *pure* common lines matrix must satisfy. Firstly, there is the following low-rank condition. All of our computational methods take advantage of this.

Theorem 3.2. *Let* 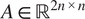 be the pure common lines matrix associated to Zariski-generic rotations 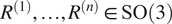 *where* 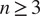 *. Then* 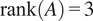 .

Proof. Since

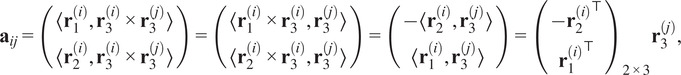

the pure commons line matrix admits the following factorization:(6)

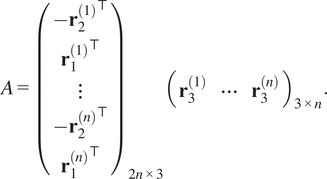

Equation (6) witnesses 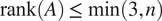 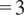 . We have equality when 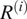 are generic because the two matrices in the factorization are full rank.

There are also necessary quadratic constraints in the entries of a pure common lines matrix.

Proposition 3.3. *Suppose* 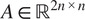 *is a pure common lines matrix where* 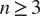 *. Then for any* 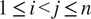 *, we have* 
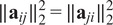
 *.*

Proof. See Appendix A.

Proposition 3.4. *Suppose* 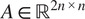 *s a pure common lines matrix where* 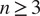 *. Then for any* 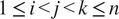 *, we have* 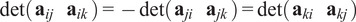 *.*

Proof. See Appendix A.

From Propositions 3.3 and 3.4, the number of quadratic constraints on a pure common lines matrix equals 
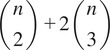
 .

Example 1. Consider 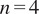 . Then a pure common lines matrix written in block form,

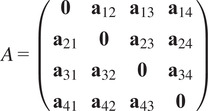

has rank at most 3 and satisfies 14 quadratic equations, which are

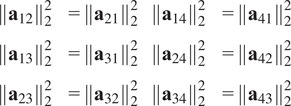




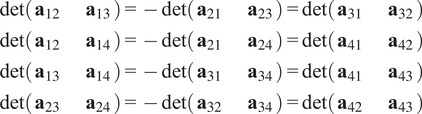

where 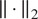 denotes the Euclidean norm. Note that 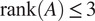 is equivalent to the vanishing of all 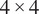 minors of 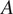 , giving a collection of homogeneous degree 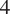 polynomial constraints on the entries of 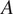 .

We note that (6) furnishes a polynomial map which sends an *n*-tuple of rotations to a pure common lines matrix:(7)

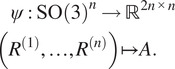

Studying 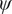 will allow us understand additional important properties of pure common lines matrices. To do this, we will need to introduce some terminology and elementary concepts from algebraic geometry (see (14) for precise definitions.)

A subset 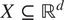 is called an *algebraic variety* if it is the set of points in 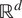 where a finite collection of polynomials all simultaneously equal 0. For example, 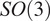 is an algebraic variety since it is the set of matrices 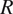 in 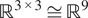 satisfying the polynomial equations 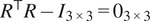 and 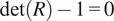 . Roughly speaking, an algebraic variety is similar to an embedded manifold, expect possibly singular and always defined by polynomial equations. Due to the properties of polynomials, an algebraic variety 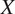 is a “thin” subset of 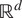 in which it lives: provided 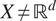 , the complement of 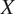 is always a dense subset filling up almost the entirety of the ambient space. More precisely, if one samples a random point from 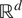 according to any absolutely continuous probability distribution, then with probability 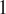 the point will lie in the complement of 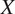 . We say that some property 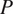 holds *(Zariski) generically* if it holds for all points in the complement of some algebraic variety 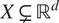 , and we call such points *(Zariski) generic.* Roughly speaking, this means that property 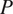 holds with probability 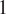 (even if, as usually the case, the variety 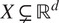 is left unspecified).

Recall that the *fiber* of a map at a point 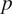 in its image is the set of points in its domain which map to 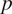 . Therefore to answer the question, “Does a pure common lines matrix uniquely determine the rotations which generated it?” we need to understand the fibers of the map 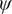 in (7). Our next result shows that the answer to the question is “Yes,” up to a global rotation, provided the pure common lines matrix is generic.

Theorem 3.5. *For* 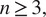 *generically, the fibers of the map* 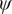 are isomorphic to 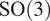 . More precisely, for generic rotations 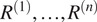 *it holds that*

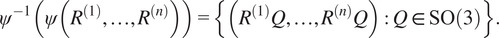

Proof. See Appendix A.

Remark 3.6. **Theorem 3.5** does not contradict the chirality ambiguity in cryo-EM, which states that the 3D molecule and rotations can only ever be recovered up to a global rotation and global reflection given cryo-EM data. In Theorem 5.1, we prove that there are two possible pure common lines matrices for a given non-pure common lines matrix. They differ by a global sign, and correspond to rotation tuples 
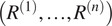
 and 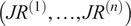 where 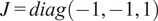 , respectively. The chirality or handedness ambiguity is well-known in the common lines literature^(^^2^^,^^5^^)^ and unavoidable.

### 3.2. The common lines variety

In general, the image of a polynomial map from an algebraic variety is not an algebraic variety, because polynomial *inequalities* (in addition to equations) are needed in the description of the image.^(^^15^^)^ This means that the set of all pure common lines matrices, that is, the image of 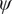 from 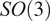 , on its own is not an algebraic variety. To resolve this, we consider the smallest algebraic variety in 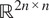 containing 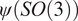 , that is, we add the smallest set of additional points (which are not pure common lines matrices) to 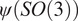 until the union becomes an algebraic variety. The process is called taking the *Zariski closure* of 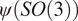 . We call the resulting algebraic variety the *common lines variety* and it lives in the ambient space 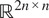 . The common lines variety is defined by polynomial equations in the entries of a matrix 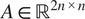 . Since the common lines variety includes all pure common lines matrices, the polynomials defining it, in particular, include the constraints we already identified in Section 3.1.

Example 2. When 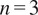 , we used the computer algebra system Macaulay2^(^^16^^)^ to determine the collection of polynomial equations defining the common lines variety. Along with the 5 quadratic polynomials from Propositions 3.3 and 3.4, our computation also found 1 polynomial of degree 6, 64 polynomials of degree 8, and 24 polynomials of degree 10, for a total of 94 polynomial equations. Notice that for 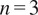 , the rank 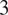 constraint of Theorem 3.2 is vacuous. We find the 5 quadratics are the only homogeneous polynomials. The other 89 equations are highly complex and we refrain from explicitly writing them here. They are available at the GitHub repository (8.1).

In view of Example 2, we believe that Section 3.1 identifies all “simple-to-describe” algebraic constraints on pure common lines matrices. As such, it is important to understand to what extent these constraints are enough to characterize pure common lines matrices. This requires understanding the geometry of the common lines variety better, for which we will need to use a couple more basic concepts from algebraic geometry described in the next two paragraphs.

In general, every algebraic variety 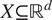 admits a unique decomposition into a finite union of *irreducible components* 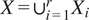 , where 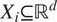 are algebraic varieties themselves and each cannot be decomposed as a union of two strictly smaller varieties. We think of 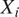 as “building blocks” of 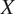 . For example, the variety 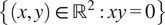 is a union of the 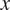 - and 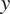 -axes, and these lines are its irreducible components. In general, each irreducible component 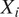 can be ascribed a *dimension*, which captures the number of degrees of freedom in 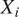 and coincides with manifold dimension when 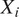 is smooth. We note that the dimension of different irreducible components 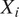 of 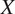 may differ.

Given an algebraic variety 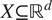 , one can construct from it a larger algebraic variety 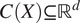 called the *cone* over 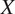 by adding to 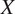 all points in 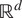 which lie on a line passing through the origin and a point on 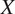 . This constructs an algebraic variety that includes all scalar multiples of points of 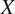 .

Since the constraints we identified in Section 3.1 are all polynomial equations, they define Section 3.1 as an algebraic variety in 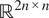 . In the next proposition, we show that this algebraic variety contains the cone over the common lines variety as an irreducible component. This means that locally on this component, our constraints from Section 3.1 are sufficient to characterize pure common lines matrices up to scale. The proof relies on computer algebra software,^(^^16^^)^ and checks that certain numerical matrices are full-rank, so we also report the range of 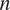 on which the proposition has been confirmed.

Proposition 3.7. *The algebraic variety defined by the low-rank constraint in Theorem 3.2, along with the quadratic constraints in Propositions 3.3 and 3.4, and the requirement that all diagonal blocks are* 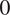 , contains the cone over the common lines variety in 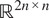 as an irreducible component for 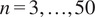 *.*

Proof. See Appendix A.

## 4. Optimization problem

We encode the common lines from cryo-EM data by choosing representatives 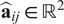 (Definition 2.1) to form the 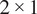 blocks in a common lines matrix 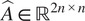 . Suppose we have rescaled the 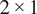 blocks of 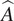 so they all have norm 1. Then at least in clean situations, Theorem 3.2 and Propositions 3.3 and 3.4 imply we can scale the blocks by nonzero scalars 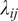 with 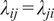 so that the resulting matrix is a *pure* common lines matrix 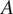 , and thus has rank 3 and satisfies the set of norm and 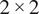 determinant equations. Proposition 3.7 states that these constraints are sufficient to determine the common lines variety locally. In Section 5.4, we further prove that for purposes of recovering scales 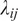 to obtain a pure common lines matrix, the constraints are also sufficient.

Proper scales are not directly available from common lines data in cryo-EM. To find the scales we formulate an optimization problem, inspired by work for a mathematically similar problem in (11):(8)

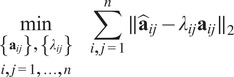


(9)

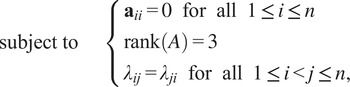


(10)

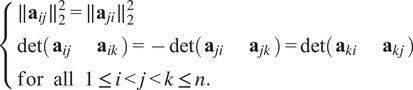



The mixed L1/Frobenius norm 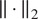 in the objective is chosen for its robustness to outliers.

Once we obtain a pure common lines matrix, we show in Section 5.3 how to recover the rotations corresponding to the common lines (up to the ambiguity in Remark 3.6). Later in Section 6, we solve the problem (8) to identify homogeneous clusters among images coming from a discrete number of distinct molecules.

## 5. Optimization algorithms

Our approach to solving (8) is first to solve the problem with constraint (9) only, and then to enforce the constraint (10) on the solution. These steps are in Sections 5.1 and 5.2, respectively.

### 5.1. IRLS and ADMM for the rank constraint

To solve (8) with the rank constraint, we closely follow the approach of (11). We relax the mixed L1/Frobenius norm to a weighted least squares objective, where the weights and optimization variables are updated after each iteration of minimization via a procedure called Iterative Reweighted Least Squares (IRLS).^(^^17^^)^ Let 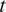 denote the IRLS iteration number. Then the objective (8) becomes:(11)

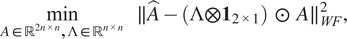

where 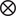 and 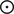 is the Kronecker and Hadamard product of two matrices respectively, 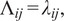




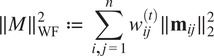

is the squared weighted Frobenius norm of a block matrix 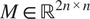 , the weights in (11) are(12)

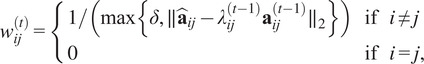

and 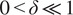 is a chosen regularization parameter.

Within each iteration of IRLS, we need to solve the problem (11) with the constraints (9). Since the objective is bilinear in 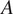 and 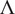 , we can do this using the Alternate Direction Method of Multiplier (ADMM).^(^^18^^,^^19^^)^ This gives an augmented Lagrangian optimization problem:(13)

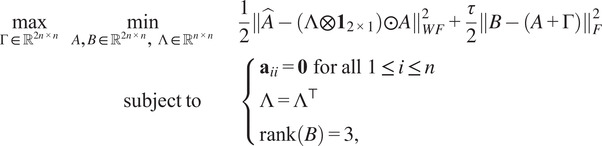

where 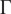 is a matrix of Lagrange multipliers and 
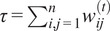
 . We now describe the steps of the ADMM procedure. Since the problem (13) is non-convex, we alternatingly optimize for each variable. In the following, let 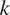 denote the ADMM iteration number and let 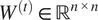 where 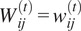 be the matrix of weights within IRLS iteration 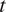 .

1. *Optimize* 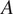 and 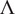 *:* We alternatingly optimize for 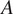 and 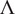 until convergence. Let 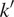 denote the iteration number for this step.
  1a. First we solve the unconstrained problem for 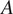 :

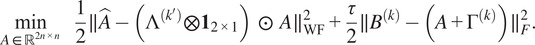

The solution is(14)

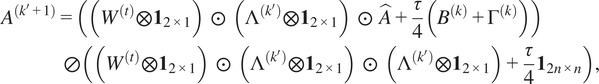

where 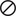 is the element-wise division of two matrices. Then we project 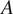 onto the set of matrices whose 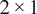 diagonals are 0:(15)

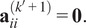

1b. Next we solve the unconstrained problem for 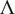 :

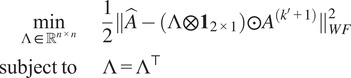

The solution is(16)

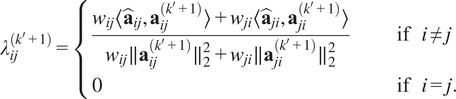

After repeating 1a. and 1b. until convergence, we obtain 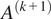 and 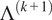 .
2. *Optimize* 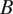 *:* The constrained problem for 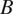 is

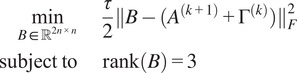

This is solved by(17)

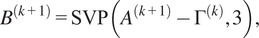

where 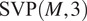 is the singular value projection of a matrix 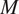 onto the set of matrices of rank at most 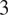 , which is computing by taking the highest three singular values of 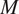 and its corresponding left and right singular vectors.
3. *Update* 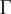 *:* In ADMM, there is a gradient ascent step for 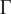 , where the step is the solution to

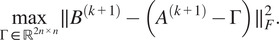

This gives the update(18)

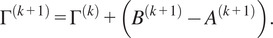

Steps 1, 2, and 3 are repeated until convergence in the optimization variables. This completes the ADMM procedure for IRLS iteration 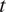 . The IRLS weights 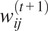 for iteration 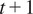 are updated using (12), and the ADMM procedure is repeated again. The whole pipeline is detailed in Algorithm 1.

Algorithm 1. **IRLS and ADMM for rank constraint satisfaction**

**Input:** 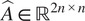 , a common lines matrix

**Output:** 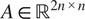 , a common lines matrix satisfying only the rank constraint

1: **procedure** IRLS-ADMM 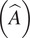

2: initialize 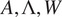

3: 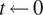

4: **while** not converged **do**

5: 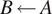

6: 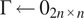

7: 
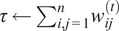

8: 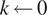

9: **while** not converged **do**

10: 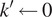

11: **while** not converged **do**

12: update 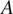 using (14) and (15) 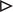 1. Update 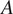 and 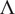

13: update 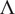 using (16)

14: 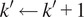

15: **end while**

16: update 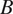 using (17) 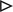 2. Update 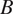

17: update 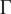 using (18) 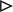 3. Update 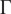

18: 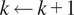

19: **end while**

20: update 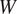 using (12) 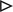 Update IRLS weights

21: 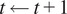

22: **end while**

23: **end procedure**

### 5.2. Sinkhorn scaling for the quadratic constraints

When successful, IRLS-ADMM in Section 5.1 gives us a solution 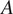 to (8) satisfying the constraint (9). Next, we must enforce (10). As described below, our approach is to scale the rows and columns of 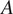 alternatingly until constraint (10) is satisfied, in a manner analogous to Sinkhorn’s algorithm.^(^^20^^)^ Note that nonzero row and column scales do not affect the rank of 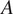 , so constraint (9) will still be satisfied.

We find the row and column scales by solving least squares problems. First, we handle the norm constraints. Define 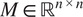 where(19)

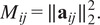



Then the norm constraints are satisfied if and only if 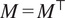 , which leads us to the following constrained least squares problems:(20)

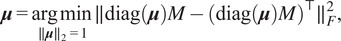


(21)

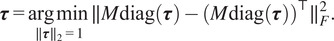

The solutions to problems (20) and (21) are(22)

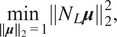


(23)

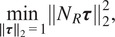

respectively, where 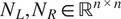 are the corresponding least squares matrices. See Appendix B for full details. The problems (22) and (23) are solved by taking 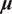 and 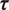 to be the right singular vector corresponding to the smallest singular value of 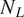 and 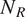 , respectively.

Now we handle the determinant constraints. Scaling each 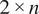 row of 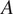 by 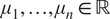 and enforcing the constraints leads us to the equations(24)

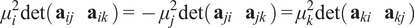

for all 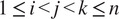 . Taking the signed root on each equation, we obtain(25)

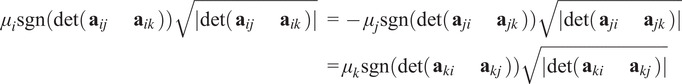

Scaling the columns of 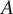 by 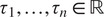 and enforcing the constraints leads to the equations(26)

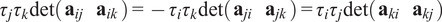

for all 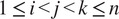 . Dividing the first equation above by 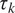 on both sides and the second equation above by 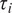 on both sides, we obtain(27)

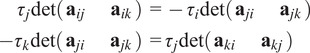

We observe that (25) and (27) are linear in 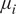 and 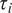 . We can enumerate all determinants into three vectors(28)

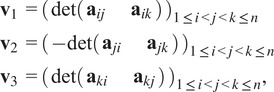

each of length 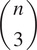 . The determinant constraints are satisfied if and only if 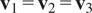 , which leads to the following constrained least squares problems:(29)

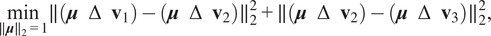


(30)

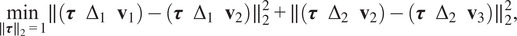

where the quantities in brackets (defined in (B5) and (B6)) are the corresponding scalings (25) and (27) of 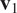 , 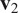 , and 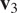 by 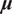 and 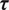 . The solutions to problems (29) and (30) are(31)

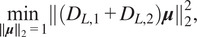


(32)

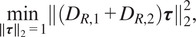

respectively, where 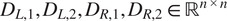 are the corresponding least squares matrices. See Appendix B for full details. The problems (31) and (32) are again solved using SVD.

Now we describe the steps of the Sinkhorn scaling method. Let 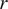 denote the iteration number of the procedure.

1. *Scale rows:* Let 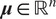 be the solution to

(33)

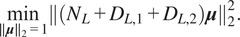

Then we perform the update(34)

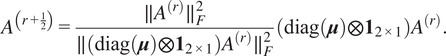

1. *Scale columns:* Let 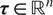 be the solution to

(35)

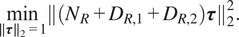

Then we perform the update(36)

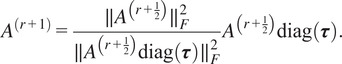

The Sinkhorn scaling procedure is detailed in Algorithm 2.

Algorithm 2. **Sinkhorn scaling for quadratic constraint satisfaction**

**Input:** 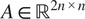 , the output of IRLS-ADMM

**Output:** 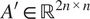 , a pure common lines matrix

1: **procedure** Sinkhorn 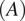

2: 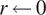

3: **while** not converged **do**

4: set 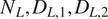 using (B1), (B7), (B8)

5: set 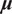 to be the solution of (33)

6: update 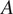 using (34) 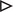 1. Scale rows

7: set 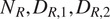 using (B2), (B9), (B10)

8: set 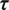 to be the solution of (35)

9: update 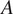 using (36) 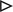 2. Scale columns

10: **end while**

11: 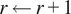

12: 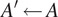

13: **end procedure**

### 5.3. Rotation recovery

IRLS-ADMM and Sinkhorn aim to output a pure common lines matrix 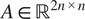 . Given a pure common lines matrix, we now show how to determine the underlying rotations 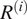 in (3) which generated 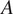 (recall Theorem 3.5).

Given 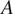 , use singular value decomposition to compute a rank-3 factorization 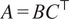 for 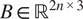 and 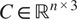 . We then seek an invertible matrix 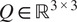 so that 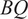 and 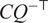 take the form of the factors in (6). In particular, it is required that the rows of 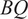 come in orthonormal pairs; more precisely, 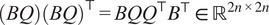 should have 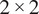 identities along its diagonal. Setting 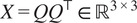 and relaxing positive semidefiniteness, let us consider the following affine-linear least squares problem:(37)

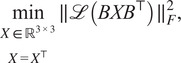

where 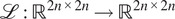 denotes the affine-linear operator which sets all entries of a 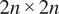 matrix outside of the 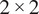 diagonal blocks to 0, and subtracts the 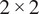 identity matrices from the diagonal blocks. The normal equations for (37) may be written as(38)

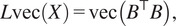

where 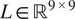 , whose rows and columns index the variables 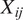 for 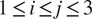 , and whose corresponding 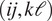 -th entry is(39)

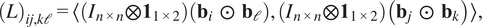

where 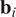 is the *i*th column of 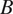 . Generically there is a unique symmetric matrix 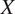 solving (38).

Let 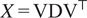 be an eigendecomposition for the solution to (37), where 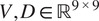 . In the clean case, the diagonal matrix 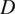 is already positive semidefinite. Then 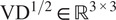 is a candidate for 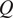 . Next the *i*th row block of 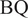 determines the first two rows of 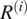 , and the cross product of these two rows computes the third row of 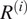 . From this, we recover the *n*-tuple of rotations 
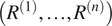
 up to global right multiplication by a rotation. The full procedure for rotation recovery is in Algorithm 3, which is formulated to apply to noisy inputs as well. We note that as explained in Remark 3.6, a pure common lines matrix 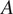 can only be recovered up to sign, because there are two distinct *n*-tuples of rotations that can be recovered corresponding to the chiral ambiguity of common lines data. One can run Rotations twice, on 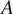 and 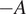 , to produce the two possible sets of rotations.

Algorithm 3. **Rotation recovery**

**Input:** 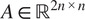 , an estimate for pure common lines matrix

**Output:** 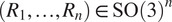 , rotations determining the pure common lines matrix

1: **procedure** Rotations 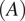

2: set 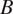 using the SVD to get a rank-3 approximation 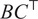 for 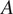

3: set 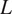 using (39)

4: set 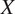 to be least squares solution of (38)

5: set 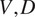 using the eigendecomposition 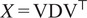

6: 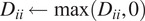

7: **for** 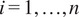 **do**

8: set 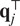 to be the 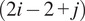 -th row of 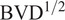 for 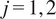

9: set 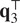 to be the *i*th row of 
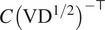

10: set 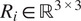 to be 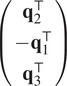

11: **end for**

12: **if** 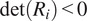 **then**

13: **for** 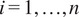 **do**

14: 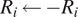

15: **end for**

16: **end if**

17: **for** 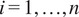 **do**

18: replace 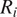 by the nearest rotation matrix to 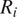 using the SVD of 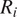

19: **end for**

20: **end procedure**

### 5.4. Justification of algorithms

Suppose the input to IRLS-ADMM is a noiseless common lines matrix 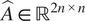 , and its output 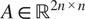 is a global minimizer to the non-convex problem (13). In the next theorem, we show that we can recover the ground-truth pure common lines matrix, up to a global scale, by scaling the 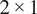 blocks of 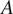 via IRLS-ADMM and enforcing the quadratic constraints via Sinkhorn. The theorem justifies using IRLS-ADMM and Sinkhorn.

Theorem 5.1. *Let* 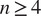 *. Let* 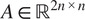 *be a generic pure common lines matrix and* 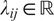 *be nonzero scales for* 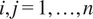 *with* 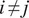 *. Let* 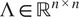 *where* 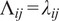 *if* 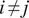 , 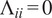 *, and* 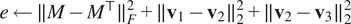 . *Suppose*


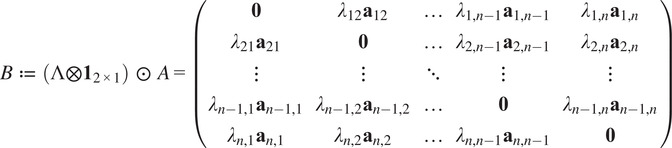

has rank 3. Then there exists 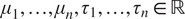 such that(40)

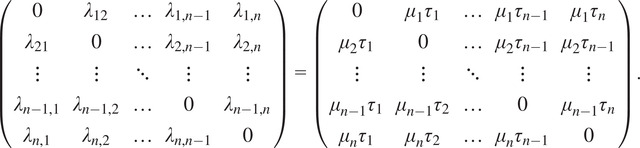


*If* 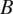 *additionally satisfies the quadratic constraints (10), then there exists* 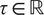 *such that for all* 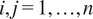 *(* 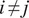 ) it holds 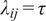 *.*

Proof. See Appendix A.

Remark 5.2. *Theorem 3.5* implies that given a pure common lines matrix, the rotation recovery problem in Section 5.3 is uniquely solvable up to a global rotation. Theorem 5.1 states that the ground-truth pure common lines matrix can only be determined up to a global scale; in particular, 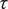 in Theorem 5.1 may be positive or negative. As in Remark 3.6, this sign flip corresponds to chiral ambiguity in cryo-EM. Apart from its sign, the global scale has no effect on rotation recovery in Algorithm 3.

## 6. Application: Clustering heterogeneous common lines

Here we present an application of our approach for common lines to a challenging problem in cryo-EM. We propose a clustering algorithm for detecting homogeneous communities of consistent common lines from discretely heterogeneous data, using our algebraic constraints. One can then use the clusters of common lines for rotation recovery and 3D reconstruction.

Several successful methods have been proposed for clustering heterogeneous cryo-EM data that consist of images of a *single* macromolecule with conformational landscapes or differences in subunits.^(^^21^^–^^27^^)^ Recent work of the third author^(^^7^^)^ proposed a method of solving a different heterogeneity challenge in cryo-EM, where the heterogeneity in the data comes from *multiple* distinct macromolecules rather than variations on one primary structure. Our proposed application focuses on the latter problem. For this setting of heterogeneity, the main prior work to compare against is (7).

**Figure 1.**
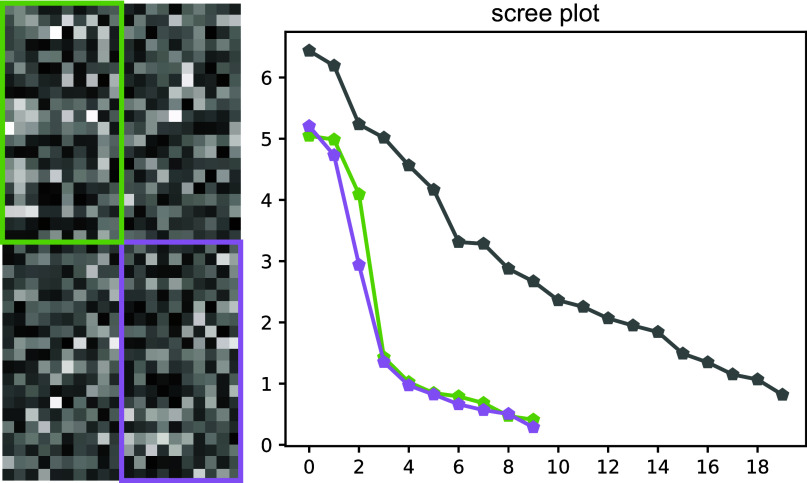
A heterogeneous common lines matrix and rank test for simulated rotations from two distinct molecules. Block-diagonals comparing rotations from the same molecule show rank-3 structure.

The basic idea of our approach is illustrated in Figure 1. There the matrix in gray is a matrix of common lines from simulated heterogeneous data, corresponding to two distinct molecular conformations. The two homogeneous common lines matrices are diagonal blocks in green and purple. The 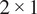 entries outside of these diagonal blocks do not correspond to any consistent lines and are just random lines in 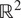 , encoded as representatives. Note that in general, a heterogeneous common lines matrix will not necessarily have consistent common lines matrices as diagonal blocks, but will be a 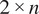 row and 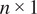 column permutation of such a matrix. Gaussian white noise has also been added to the gray matrix to decrease the signal-to-noise ratio of the common lines. A scree plot in Figure 1 shows a noticeable spectral gap between the third and fourth singular values of the homogeneous common lines matrices (green and purple curve), which is not detectable for the entire heterogeneous common lines matrix (gray curve), thus demonstrating low-rank structure of submatrices.

Algorithm 4. Heterogeneous clustering on common line constraints

**Input:** 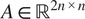 , a common lines matrix

**Output:** 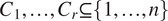 , a partition of all common lines into consistent clusters

1: **procedure** Clusters 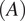

2: 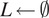 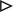 1. Generate samples

3: **for** 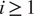 until sufficient **do**

4: set 
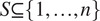
 to be a random sample such that 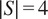

5: set 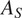 to be the submatrix of 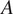 associated to the common lines in 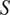

6: *A_S_*←Irls-Admm (*A_S_*)

7: *A_S_* ← Sinkhorn (*A_S_*)

8: **if** converged **then**

9: set 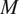 using (19) on 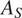

10: set 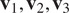 using (28) on 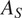

11: 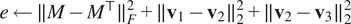

12: 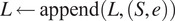

13: **end if**

14: **end for**

15: sort 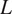 by increasing values of 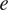 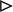 2. Cluster samples

16: 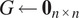

17: **while** 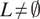 **do**

18: 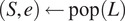

19: 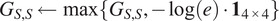

20: **end while**

21: 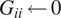 for all 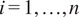

22: *C*_1_,…,*C_r_* ← CommunityDetection(*G*)                                             ⊳ Use method from (28)

23: **end procedure**

Our clustering algorithm consists of two main steps:

1. *Generate samples:* As input, we are given a single common lines matrix 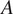 , from which we identify small clusters of consistent common lines. We randomly sample a set of four common lines 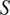 and extract the corresponding 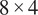 submatrix 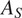 from 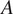 (the choice of four common lines is explained in Section 7.3 based on numerical experiments). We then run IRLS-ADMM and Sinkhorn on 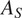 . These methods may occasionally diverge due to numerical instability from noise in the data, as discussed in Section 7, in which case we discard the sample. We also discard the sample if the spectral gap between the third and fourth singular value of 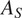 is not sufficiently large (i.e., 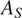 does not have numerical rank 3). Otherwise, we obtain a quadratic constraint satisfaction error for the sample. We record both the sample and its error, and repeat this sampling sufficiently many times.
2. *Cluster samples*: We view the collection of samples and errors we obtain as a weighted hypergraph on 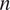 vertices whose hyperedges are of size 4 corresponding to the samples and whose hyperedge weights are given by their corresponding errors. We convert the weighted hypergraph into a weighted graph by constructing a weighted adjacency matrix whose 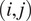 entry is the negative logarithm of the smallest error on a hyperedge containing both common lines 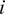 and 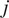 . We then use an unsupervised community detection algorithm on this adjacency matrix to find the clusters of consistent common lines. In our numerical experiments, we use the algorithm proposed by Lancichinetti, Fortunato, and Kertész,^(^^28^^)^ which can identify overlapping communities and hierarchical structure, and depends only on a single hyperparamter controlling the scale of the hierarchies. In particular, we do not need to specify the number of clusters or their sizes.

**Figure 2.**
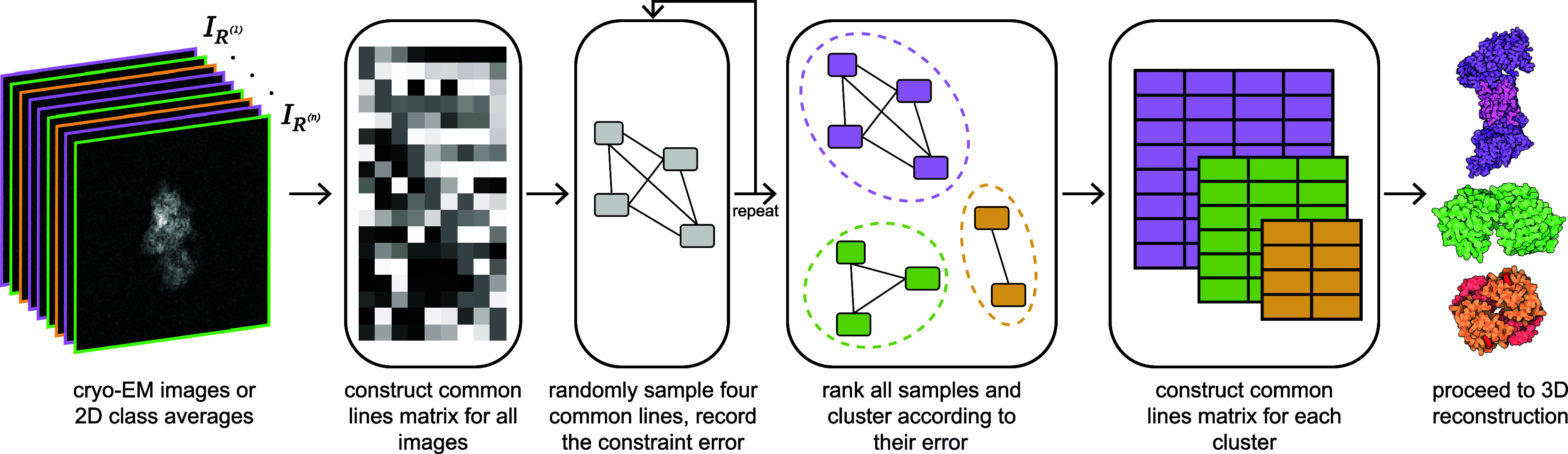
Algorithm for separating images of distinct molecules using algebraic constraints on common lines. The common lines matrix is first computed from an input set of images or class averages. We then apply Algorithm 4, our clustering algorithm. After clustering, images corresponding to the same molecule can then be used for 3D reconstruction.

The clustering algorithm is detailed in Algorithm 4 and illustrated in Figure 2.

## 7. Performance on data

We compare the performance of our methods to existing common lines based algorithms in the literature, namely functions in the software package ASPIRE ^(^^29^^)^ and the clustering algorithm of Verbeke et al.^(^^7^^)^ We study the problems of recovering rotations, denoising common lines, and partitioning discretely heterogeneous image sets into homogeneous subcommunities using common lines. The tests are done on simulated data (at various levels of noise) and real data.

**Figure 3.**
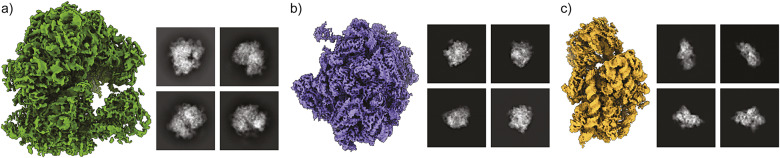
3-D structures and example projection images for the three structures used for simulation. (a) 80S ribosome (EMD-2858) and example projection images. (b) 60S ribosome (EMD-2811) and example projection images. (c) 40S ribosome (EMD-4214) and example projection images.

Our simulated data consists of image data of the 40S, 60S, and 80S ribosome (available from the Electron Microscopy Data Bank^(^^30^^)^ as entries EMD-4214, EMD-2811, and EMD-2858, respectively), generated by ASPIRE. The ribosomes and examples of their clean 2D projection images are displayed in Figure 3. Each image is 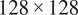 with a pixel size of 3 Å. White Gaussian noise is added to each image corresponding to a specified signal-to-noise ratio (SNR). We define the SNR by taking the signal to be the average squared intensity over each pixel in the clean image and setting the noise variance to achieve the appropriate ratio. The common lines between two images are detected by finding the line projections of the two images with the highest correlation, as computed in ASPIRE.

In our numerical experiments, we have observed that the performance of IRLS-ADMM, and consequently Sinkhorn, can depend on its initialization. In particular, we have occasionally observed divergence or vanishing of the entries of 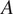 in Sinkhorn. This behavior appears to be due to numerical instabilities in Sinkhorn arising from noise in the data or from using too many common lines. In these cases, we can either discard such runs and restart the algorithms with new initializations, or skip using Sinkhorn. We address this issue in each of our tests.

### 7.1. Rotation recovery

Let 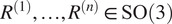 be the ground-truth rotations and 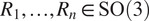 be the recovered rotations. Then Theorem 3.5 states that there exists a unique rotation 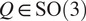 such that 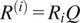 for all 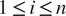 . In other words, 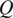 can be found by solving the orthogonal Procrustes problem(41)

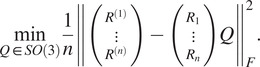

The solution to this problem can be found using SVD.^(^^31^^)^ We note that for 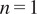 , there is a simple relation between the Procrustes error and the angular error between two rotation matrices. For 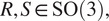 it holds 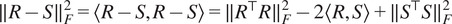 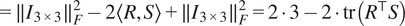 . Hence the angular error between 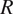 and 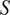 is

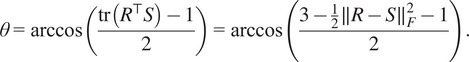



We compare our method to the procedure in ASPIRE on simulated data. Given common lines, the rotation recovery algorithm used in ASPIRE is the synchronization with voting procedure as described in (5). We use 30 images of the 60S, 80S, and 40S ribosomes at 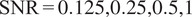 . For each macromolecule and SNR, we generate 50 sets of 30 random ground-truth rotations and their corresponding noisy images. We report the average Procrustes error (41) per image (i.e., the Procrustes error divided by the number of images).

When running this test, we chose to only use our IRLS-ADMM algorithm followed by Rotations, and omitted the Sinkhorn row and column scaling step due to observed numerical instabilities of Sinkhorn with noise in the data or too many common lines. Also, Remark 5.2 states that our algorithm is only guaranteed to recover the ground-truth pure common lines matrix up to a global scale, and the sign of this scale produces two sets of rotations that differ by left-multiplication with 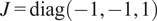 . This sign flip corresponds to the chiral ambiguity in cryo-EM, as explained in Remark 3.6. Thus in our tests, we report the best rotational error amongst the two possible sets of rotations.

The results for rotation recovery are displayed in Table 1. Notably, at lower SNR our method is consistently more accurate than ASPIRE.

**Table 1.** The average rotation recovery error from 30 simulated images of macromolecules at various SNR, 50 runs each

		Average Procrustes rotation error (42)	
Macromolecule	SNR	Algorithm 1 and Algorithm 3	ASPIRE
EMD–2811 (60S ribosome)	0.125	**3.6840**	4.3426
	0.25	**3.3215**	3.6042
	0.5	**1.5512**	2.2704
	1	**0.4215**	0.4877
EMD–2858 (80S ribosome)	0.125	**1.7903**	2.5943
	0.25	**0.8038**	1.3175
	0.5	**0.1576**	0.2543
	1	0.0259	**0.0240**
EMD–4214 (40S ribosome)	0.125	**4.0377**	4.5281
	0.25	**3.9079**	4.0104
	0.5	3.6375	**3.3994**
	1	2.8271	**2.1334**

Bold values indicate the algorithm with lower error.

### 7.2. Denoising common lines

The problem we consider here is the following: given a *noisy* common lines matrix, how well can we recover the ground-truth clean pure common lines matrix? If 
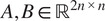
 are the ground truth and recovered pure common lines matrix respectively, then we measure this error to be(42)

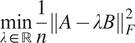

since there is a global scale ambiguity in the recovered pure common lines matrix as discussed in Remark 5.2. The above problem is a least-squares problem in 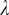 and hence has a closed-form solution.

We compare our methods to ASPIRE on simulated data as follows. We run the rotation recovery algorithm of ASPIRE based on common lines to obtain rotations 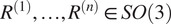 . Then we construct a recovered pure common lines matrix 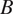 from these rotations by using the factorization (6). We then compare the denoising error (42) from the ground-truth clean common lines matrix 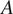 to the output of our IRLS-ADMM method and to the matrix 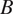 . As before, the simulated data consists of 30 images of the 60S, 80S, and 40S ribosomes at 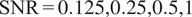 , and we report the average denoising error (42) per image over 50 runs. We assume we have the ground-truth chiral information when recovering rotations with ASPIRE.

The results for common line denoising are displayed in Table 2. Again our IRLS-ADMM method outperforms ASPIRE for denoising common lines matrices, particularly at low SNR.

**Table 2.** The average denoising error of recovered pure common lines matrices from 30 simulated images of macromolecules at various SNR, 50 runs each

		Average denoising error (42)	
Macromolecule	SNR	Algorithm 1	ASPIRE
EMD–2811 (60S ribosome)	0.125	**14.1982**	18.2274
	0.25	**12.7786**	17.8805
	0.5	**8.2349**	16.8199
	1	**4.1908**	6.1365
EMD–2858 (80S ribosome)	0.125	**11.2948**	17.3292
	0.25	**6.8850**	13.3778
	0.5	**2.3152**	3.6690
	1	0.5680	**0.3512**
EMD–4214 (40S ribosome)	0.125	**15.5635**	18.0612
	0.25	**15.1112**	18.0862
	0.5	**14.0420**	18.0069
	1	**11.6964**	15.7237

Bold values indicate the algorithm with lower error.

### 7.3. Clustering heterogeneous image sets

We test the performance of our algorithm Clusters for clustering (see Section 6) on simulated and real data.

The success of clustering is measured by the adjusted Rand index^(^^32^^)^ (ARI) between the ground-truth clusters and the recovered clusters. The range of this index is 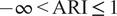 , with 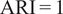 if the two partitions are identical. The ARI is a corrected-for-chance version of the Rand index, meaning that it is the expected Rand index for the cluster and is equal to 0 if every element is placed in a random cluster.

While any number of common lines 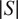 can be sampled on line 4 of Clusters, we found that sampling four common lines at a time was effective for a number of reasons: 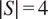 enforces a non-trivial rank 3 constraint, and the small number of common lines allowed us to both generate many samples rapidly and improve the numerical stability of the Sinkhorn scaling procedure. In addition, the CommunityDetection algorithm we use is the one described in (28).

#### 7.3.1. Simulated data

We generate a dataset containing three clusters, with 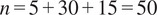 images from the 40S, 60S, and 80S ribosomes, respectively, from which we construct a common lines matrix.

**Figure 4.**
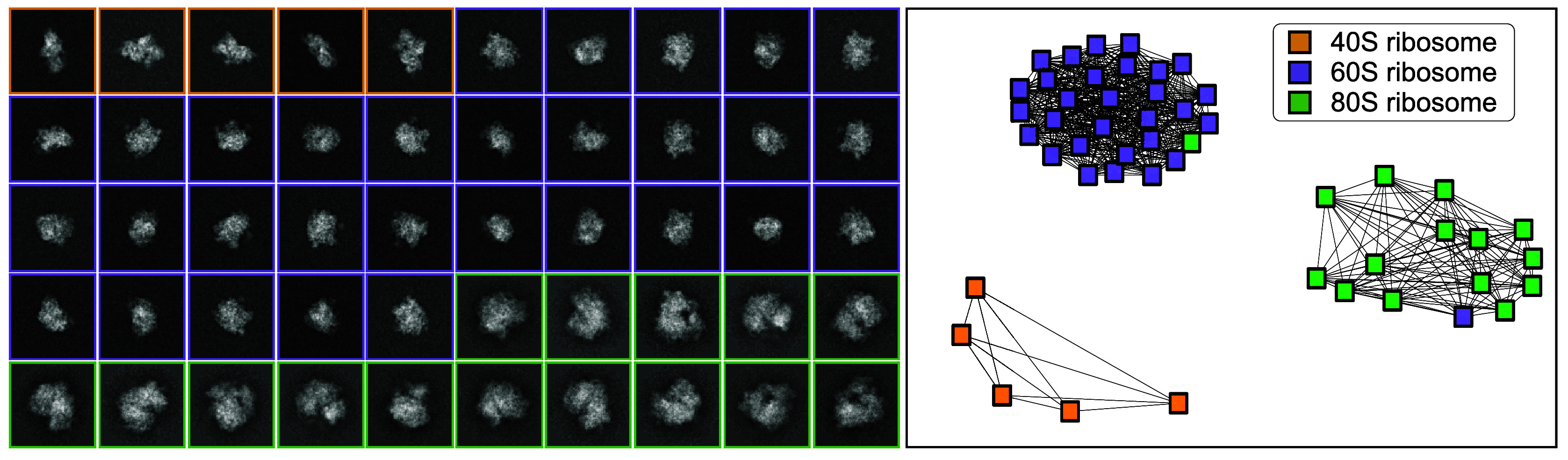
Clustering results for 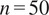 simulated images with SNR = 5. Images are size 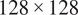 with pixel size of 3 Å, and are colored according to the ground truth labels. Using Clusters achieves ARI = 0.8581 and only one pair of images are incorrectly clustered.

Clusters achieved perfect clustering ( 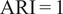 ) at 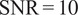 , 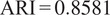 at 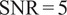 , and 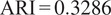 at 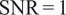 . The clusters found at 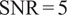 are displayed in Figure 4, which shows that only one pair of images were placed in incorrect clusters.

#### 7.3.2. Real data

Our real data consists of a subset of 2D class averages computed from the experimental data described in Verbeke et al.^(^^7^^)^ The subset we consider consists of two clusters with 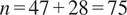 images corresponding to the 60S and 80S ribosomes respectively. Each class average is 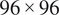 with a pixel size of 4.4 Å. We use the labels from (7) as ground truth for clustering.

**Figure 5.**
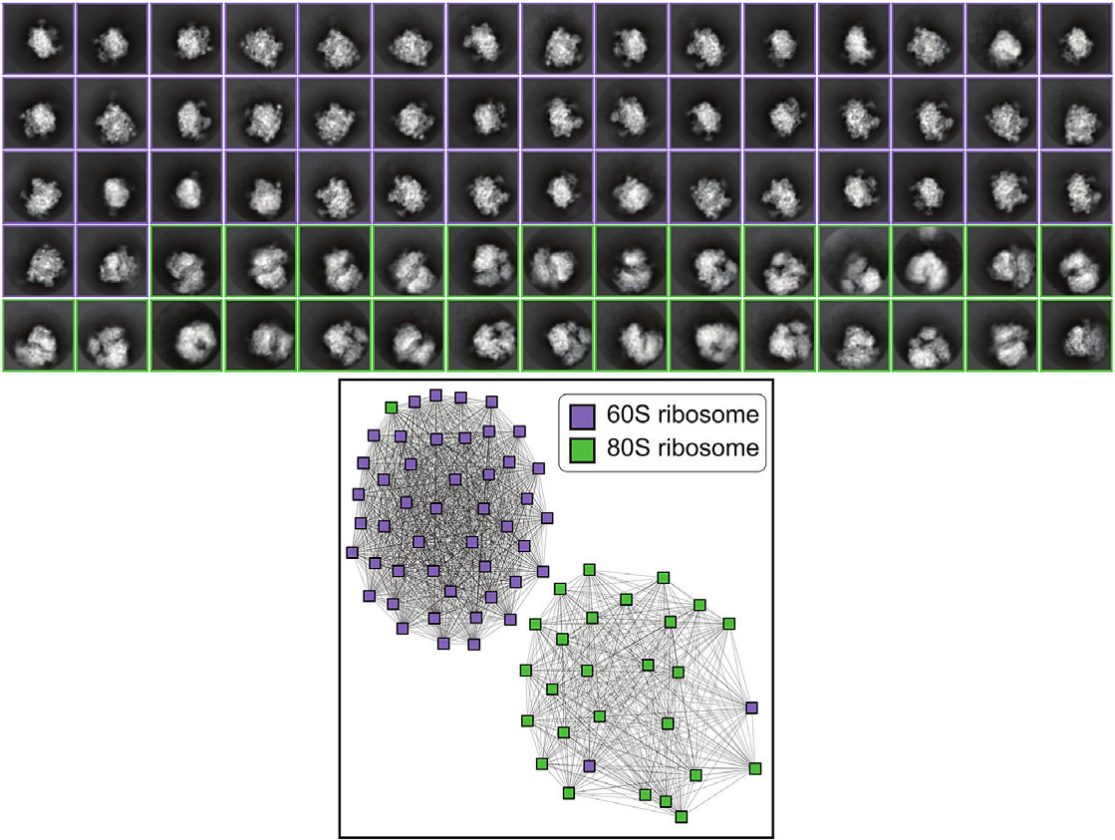
Clustering results for 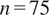 2D class averages from EMPIAR-10268 computed as described in (7). Images are size 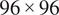 with a pixel size of 4.4 Å, and are colored according to the ground truth labels. Using Clusters achieves ARI = 0.8440 and only three images are incorrectly clustered.

Figure 5 shows the clusters found by our algorithm Clusters, achieving ARI = 0.8440 and misclassifying only three images.

The clustering algorithm used in (7) is based on performing community detection on a nearest-neighbors graph constructed using *Euclidean distances* between the best-matching line projections between every pair of images. We stress that our clustering algorithm uses a completely distinct aspect of common lines data: the *positions* of the common lines. As a proof of concept for our constraints, we do not make use of the correlations between the common lines at all, unlike (7). The test for Clusters only uses a subset of the dataset in (7), which has 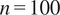 images and includes images with unknown labels. If we compare only the 60S and 80S images that were clustered, then Clusters achieves a similar performance, where one additional image is misclassified by Clusters compared to (7).

## 8. Conclusion

This article revisited the fundamental topic of common lines in cryo-EM image processing. We discussed a novel approach for dealing with common lines, based on a certain 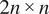 matrix encoding the common lines between 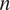 projection images. We proved that if the 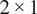 blocks of the matrix are properly scaled, then the matrix satisfies nice algebraic constraints: a low-rank condition and several sparse quadratic constraints. The new formulation operates directly on common lines data, and is fully global in that it does not require angular reconstitution or voting procedures at all. It opens the door to different and potentially more robust approaches to computational tasks involving common lines. Using the algebraic constraints, we adapted optimization algorithms from other domains to give new methods to denoise common lines data, and recover the 3D rotations underlying noisy images. Numerical experiments show that these methods have increased accuracy at low SNR, compared to existing methods based on common lines. We also explored a setting where traditional common lines methods fail to apply – cryo-EM datasets with discrete heterogeneity – by proposing a sampling-based process to cluster the images in homogeneous subcommunities based on our algebraic constraints. Experiments with simulated and real data show the method performs well when applied to images with high noise.

Although there is clear promise, several future directions could be pursued for further improvements. Firstly, in Section 5 the optimization algorithm building on (11) is quite complex. Matrix scaling problems as in (11) and our work are an interesting variation on the problem of matrix completion; would other optimization approaches perform better? Secondly, extensions to molecules with nontrivial point group symmetries would be useful (and currently are a focus in other common lines research). Perhaps our formulation can suggest another way to incorporate symmetries into common lines methods. Lastly, in the application to discrete heterogeneity, we neglected correlation scores between the common lines, on which (7) relied. It is likely better to use both the scores and the algebraic constraint errors.

## Data Availability

Data and replication code are available at https://github.com/ozitommi/algebraic-common-lines.

## A. Appendix. Additional proofs

Proof of Proposition 3.3. Since orthogonal matrices preserve norms, we have

(A1)

Similarly, we have

(A2)

Thus, by equating (A1) with (A2) and simplifying, we obtain  .
Proof of Proposition 3.4. Let  . Then for each  we have

(A3)

by expanding the determinant along the first column. Similarly for each  and  we have

(A4)

(A5)

Thus, equating (A3)–(A5), we obtain  .
Proof of Theorem 3.5. We first prove the theorem in the case  . Given  , we have  for any  since

Hence, the fibers of  contain at least a copy of  By fixing  we may assume that  so that  . To prove the statement, we therefore need to show that fibers of the map  with  fixed,

(A6)

generically consist of only one point. Our strategy to show this is to set up a corresponding system of polynomial equations for a random instance of two rotations  and  , and then solve the system numerically, showing that it has only one solution. It is sufficient to solve the polynomial system over the complex numbers  , and exhibit that there is a unique solution even over  . We parameterize  using the Euler-Rodriguez formula:

where  such that  . We construct one pure common lines matrix  whose entries are polynomial in  , and another pure common lines matrix  , from random rotation matrices  , whose entries are real. Setting  gives us a system of polynomial equations, which we solve over  using the package HomotopyContinuation.jl in Julia.^(^^33^^)^ Every rotation under the Euler-Rodriguez parametrization has two parametrizations, namely  and  representing the same matrix in  . By parameterizing  and  , we therefore expect to find 4 real solutions to the system and therefore one point in the fiber, which is indeed what we find using homotopy continuation. The computation is carried out in the file fiber.jl in our GitHub repository (8.1). Now with the theorem true for  , it in fact follows that the theorem is true for all  : if  and  , where  is a generic point in the image of  , then we need to show that there exists a unique  such that  for all  . Choosing two  pure common lines submatrices of  corresponding to indices  and  , the theorem in the case  implies that there exist  such that  and  . In particular,  , so  . Thus, any triplet of rotations are related by the same matrix  .
Proof of Proposition 3.7. Let  denote the cone over the common lines variety (i.e.,  is the smallest algebraic variety containing all scalar multiples of all pure common lines matrices). Let  denote the variety defined by the polynomial constraints in Proposition 3.7. Note that since the constraints are invariant to global scaling, we have  . By (15), in order to show that  is an irreducible component of  we need to show that

(A7)

holds a generic point  , where  and  respectively denote the dimension of a variety and the local dimension of a variety at a point. This is analogous to computing the dimension of a manifold by computing the dimension of its tangent space at a point. Recall  is the cone over the common lines variety, which is the smallest algebraic variety containing the image of the map  in (7). Thus using the fiber dimension theorem (the algebraic geometric analog of the rank-nullity theorem from linear algebra), Theorem 3.5 implies

(A8)

Next, we compute  . We first note that  , where  is the algebraic variety of all rank  matrices in  defined by the vanishing of  minors. Note the map  given by  parameterizes  with  -dimensional fibers over each rank-3 point.^(^^34^^)^ This holds because for each  invertible matrix  ,  . Hence

(A9)

for  such that  . We now compute the right-hand side of (A9) in the computer algebra system Macaulay2.^(^^16^^)^ Specifically, we differentiate the defining constraints of  with respect to  and  , noting that the rank-3 constraint is enforced automatically by  and the other constraints in Proposition 3.7 are biquadratic and bilinear in  . We evaluate the Jacobian matrix at a point  in the fiber of  over  , where  is generated as a random scaling of  where  are random. Generically, the nullity of the Jacobian matrix equals  . Performing numerical computations for  in double-precision arithmetic yields numerical nullities of  . Comparing (A9) with (A8) implies (A7) as desired.
Proof of Theorem 5.1. Since nonzero row and column scaling preserves rank, we may assume without loss of generality that  for all  : this is achieved by choosing  ,  , and  . We will show that  for all  ,  ,  . In other words, we will prove that the matrix  must have the form in (A12). First, we prove that  for all  ,  ,  . There are three cases to consider:  ,  , and  . Suppose we are in the first case. Since  every  minor of  vanishes. Thus, choosing row indices 1, 2,  , and  , and column indices 1,  ,  , and  , we have

(A10)

But since  is a pure common lines matrix,  and det  by Proposition 3.4, all of which are nonzero since  is generic. Thus  . Expanding the  minor with the same choice of row and column indices for the remaining two cases gives us

and

respectively, which brings us back to (A10). Suppose that  . The  minor whose row indices are  ,  ,  , and  , and whose column indices are 1,  ,  , and  is

We showed earlier that  and  . Thus expanding, we obtain

(A11)

After dividing both sides of the last equation by  , we obtain  . Lastly, the  minor with the same choice of row and column indices when  is

since  and  , which brings us back to (A11). Thus we have proven that  for all  ,  ,  . Let  be their common value. Then

(A12)

and we may obtain (40) by choosing  ,  and  ,  . Now we prove the last statement of the theorem. What we have shown so far is that  implies that there exists scales  and  such that  . Since  , we have

for all  . If  and  , then this means that the matrix  is symmetric. This is true if and only if  . In particular,

(A13)

If  furthermore satisfies the determinant constraints, then we have

for all  , which implies that

Substituting (A13), we obtain

after dividing by  . Denoting the common value of the above equation on the right by  , we find that  . Hence,  for all  ,  , which gives the desired result.

## B. Appendix. Least squares problems

In Section 5.2, we consider the least squares problems (20) and (21), which are

These have solutions

respectively, where  with

(B1)

and  with

(B2)

We also consider the least squares problems (29) and (30), which are

(B3)

(B4)

where

(B5)

and

(B6)

are all vectors of length  . The solution to problem (B3) is

where  with

(B7)

(B8)

and the solution to problem (B4) is

where  with

(B9)

(B10)

where  .
